# Differences in Inflammatory Pathways Between Dutch South Asians vs Dutch Europids With Type 2 Diabetes

**DOI:** 10.1210/clinem/dgac598

**Published:** 2022-10-20

**Authors:** Maaike E Straat, Borja Martinez-Tellez, Huub J van Eyk, Maurice B Bizino, Suzanne van Veen, Eleonora Vianello, Rinke Stienstra, Tom H M Ottenhoff, Hildo J Lamb, Johannes W A Smit, Ingrid M Jazet, Patrick C N Rensen, Mariëtte R Boon

**Affiliations:** Division of Endocrinology, Department of Medicine, Leiden University Medical Center, 2333 ZA Leiden, the Netherlands; Einthoven Laboratory for Experimental Vascular Medicine, Leiden University Medical Center, 2333 ZA Leiden, the Netherlands; Division of Endocrinology, Department of Medicine, Leiden University Medical Center, 2333 ZA Leiden, the Netherlands; Einthoven Laboratory for Experimental Vascular Medicine, Leiden University Medical Center, 2333 ZA Leiden, the Netherlands; Division of Endocrinology, Department of Medicine, Leiden University Medical Center, 2333 ZA Leiden, the Netherlands; Einthoven Laboratory for Experimental Vascular Medicine, Leiden University Medical Center, 2333 ZA Leiden, the Netherlands; Division of Endocrinology, Department of Medicine, Leiden University Medical Center, 2333 ZA Leiden, the Netherlands; Department of Radiology, Leiden University Medical Center, 2333 ZA Leiden, the Netherlands; Department of Infectious Diseases, Leiden University Medical Center, 2333 ZA Leiden, the Netherlands; Department of Infectious Diseases, Leiden University Medical Center, 2333 ZA Leiden, the Netherlands; Nutrition, Metabolism and Genomics Group, Division of Human Nutrition and Health, Wageningen University, 6708 PB Wageningen, the Netherlands; Department of Medicine, Radboud University Medical Center, 6525 XZ Nijmegen, the Netherlands; Department of Infectious Diseases, Leiden University Medical Center, 2333 ZA Leiden, the Netherlands; Department of Radiology, Leiden University Medical Center, 2333 ZA Leiden, the Netherlands; Department of Medicine, Radboud University Medical Center, 6525 XZ Nijmegen, the Netherlands; Division of Endocrinology, Department of Medicine, Leiden University Medical Center, 2333 ZA Leiden, the Netherlands; Einthoven Laboratory for Experimental Vascular Medicine, Leiden University Medical Center, 2333 ZA Leiden, the Netherlands; Division of Endocrinology, Department of Medicine, Leiden University Medical Center, 2333 ZA Leiden, the Netherlands; Einthoven Laboratory for Experimental Vascular Medicine, Leiden University Medical Center, 2333 ZA Leiden, the Netherlands; Division of Endocrinology, Department of Medicine, Leiden University Medical Center, 2333 ZA Leiden, the Netherlands; Einthoven Laboratory for Experimental Vascular Medicine, Leiden University Medical Center, 2333 ZA Leiden, the Netherlands

**Keywords:** B lymphocytes, ethnicity, gene expression, interferon gamma, metabolic syndrome

## Abstract

**Context:**

South Asian individuals are more prone to develop type 2 diabetes (T2D) coinciding with earlier complications than Europids. While inflammation plays a central role in the development and progression of T2D, this factor is still underexplored in South Asians.

**Objective:**

This work aimed to study whether circulating messenger RNA (mRNA) transcripts of immune genes are different between South Asian compared with Europid patients with T2D.

**Methods:**

A secondary analysis was conducted of 2 randomized controlled trials of Dutch South Asian (n = 45; age: 55 ± 10 years, body mass index [BMI]: 29 ± 4 kg/m^2^) and Dutch Europid (n = 44; age: 60 ± 7 years, BMI: 32 ± 4 kg/m^2^) patients with T2D. Main outcome measures included mRNA transcripts of 182 immune genes (microfluidic quantitative polymerase chain reaction; Fluidigm Inc) in fasted whole-blood, ingenuity pathway analyses (Qiagen).

**Results:**

South Asians, compared to Europids, had higher mRNA levels of B-cell markers (*CD19*, *CD79A*, *CD79B*, *CR2*, *CXCR5*, *IGHD*, *MS4A1*, *PAX5*; all fold change > 1.3, false discovery rate [FDR] < 0.008) and interferon (IFN)-signaling genes (*CD274*, *GBP1*, *GBP2*, *GBP5*, *FCGR1A/B/CP*, *IFI16*, *IFIT3*, *IFITM1*, *IFITM3*, *TAP1*; all FC > 1.2, FDR < 0.05). In South Asians, the IFN signaling pathway was the top canonical pathway (*z* score 2.6; *P* < .001) and this was accompanied by higher plasma IFN-γ levels (FC = 1.5, FDR = 0.01). Notably, the ethnic difference in gene expression was larger for women (20/182 [11%]) than men (2/182 [1%]).

**Conclusion:**

South Asian patients with T2D show a more activated IFN-signaling pathway compared to Europid patients with T2D, which is more pronounced in women than men. We speculate that a more activated IFN-signaling pathway may contribute to the more rapid progression of T2D in South Asian compared with Europid individuals.

South Asian individuals, originating from the Indian subcontinent and encompassing 20% of the world population, are at particularly high risk of developing type 2 diabetes (T2D). In high-income countries such as the Netherlands, South Asian people have a 2- to 4-times higher risk of developing T2D compared to people of European origin, in the manuscript further called “Europid” (1). Notably, at the moment of diagnosis, a high proportion of South Asian patients with T2D have a lower body mass index (BMI) and are at a younger age as compared to Europids. Additionally, in South Asians microvascular and macrovascular complications of T2D evolve within a shorter duration after start of the disease (2, 3). Several factors have been proposed to underlie the increased susceptibility of South Asians to develop T2D compared to Europids, such as genetic predisposition, differences in lifestyle, central adiposity, low lean mass, low brown adipose tissue volume, and insulin resistance (4, 5). However, the high rate of T2D in South Asian individuals cannot be fully explained by these factors alone.

Over the last decades, inflammation has been increasingly acknowledged to play an important role in the pathogenesis of T2D, at least partly by accelerating insulin resistance (6–8). Accordingly, clinical studies have shown that anti-inflammatory treatments, such as the interleukin-1-receptor antagonist Anakinra and the anti-inflammatory compound salsalate, improve glycemic control in patients with T2D (9–12). Although anti-inflammatory therapy is thus regarded as a promising strategy to improve T2D regulation, these studies have so far been conducted only in patients of Europid origin. Interestingly, previous studies support the presence of a more proinflammatory phenotype in South Asians compared with Europids. Concentrations of the nonspecific inflammatory marker C-reactive protein are higher in healthy middle-aged South Asian compared with Europid men and women (13, 14). Furthermore, interleukin-6 levels are higher in healthy young South Asian men (15) and healthy middle-aged South Asian women than in matched Europids (16). These data thus support a possible pathophysiological role for inflammation in explaining the increased risk of the South Asian population to develop T2D. However, which aspects of the immune system may be differentially regulated in South Asians with T2D is currently unknown.

To further pinpoint such a role and to elucidate the possible clinical benefit of anti-inflammatory therapy to reduce T2D burden in the South Asian population, a detailed overview of the inflammatory state of South Asian patients with T2D is urgently needed. Therefore, the aim of this study was to investigate whether circulating messenger RNA (mRNA) transcripts of a broad range of immune related genes (using an untargeted approach by measuring, eg, markers of T cells, B cells, natural killer cells, and interleukins) are different between patients with T2D from Dutch South Asian vs Dutch Europid descent.

## Materials and Methods

### Study Design and Participants

The present study is a secondary analysis of 2 previously performed double-blind, placebo-controlled, randomized clinical trials that were both designed to investigate the effect of 26-week liraglutide treatment on cardiovascular end points in overweight and obese patients with T2D (17, 18). In the first trial (performed 2013-2016), 49 Dutch Europid patients with T2D were included (17). In the second trial (performed 2015-2018), 47 Dutch South Asian patients with T2D were included, of whom South Asian ethnicity was based on being born and raised in the Netherlands and having 4 grandparents from South Asian descent (18). Due to missing samples, in the present study 44 Dutch Europid (19 women; 43%) and 45 Dutch South Asian (27 women; 60%) patients with T2D were included. For both trials, inclusion criteria were BMI greater than or equal to 23; age 18 to 74 years; and glycated hemoglobin A_1c_ (HbA_1c_) greater than or equal to 6.5% and less than or equal to 11.0% (≥ 47.5 and ≤ 96.4 mmol/mol). Patients were allowed to be treated with glucose-lowering medication (exclusively metformin, sulfonylurea derivatives, and insulin), although with a stable dosage for at least 3 months before participation in the study. Patients were allowed to use antihypertensives and statins. Exclusion criteria were use of glucose-lowering medication other than those mentioned earlier; presence of renal disease; congestive heart failure according to New York Heart Association classification III to IV; uncontrolled hypertension (systolic blood pressure > 180 mm Hg and/or diastolic blood pressure > 110 mm Hg); or an acute coronary or cerebrovascular accident within 30 days before study inclusion. The trials were conducted at the Leiden University Medical Center, the Netherlands, and were approved by the local ethics committee. Written informed consent was obtained from all individuals before inclusion. The trials were conducted in accordance with the principles of the revised Declaration of Helsinki and were registered at clinicaltrials.gov (NCT01761318 and NCT02660047, respectively).

### Study Procedures

This is a cross-sectional, study. The data used for the present secondary analyses were obtained at baseline before patients started their liraglutide treatment. During this visit, participants fasted for at least 6 hours. Their medical history was consulted to, among other things, obtain information about their diabetes duration and medication use. Body weight and total fat mass were assessed using bioelectrical impedance analysis (Bodystat 1500, Bodystat Ltd). Visceral adipose tissue (VAT) mass and abdominal subcutaneous adipose tissue (SAT) mass were assessed by magnetic resonance imaging (MRI).

### Magnetic Resonance Imaging Protocol

The MRI protocol has been described in detail previously (19). In short, all participants underwent an MRI using a clinical 3 Tesla Ingenia whole-body MR system (Philips Medical Systems) at baseline and after 26 weeks of liraglutide treatment. Participants were scanned in the supine position after at least 6 hours fasting. Semiautomated segmentation of VAT and abdominal SAT was depicted by threshold-based inclusion of fat, with manual correction. VAT and SAT were calculated as mean area of fat in 3 slices.

### Blood Samples

Venous blood samples were drawn from the antecubital vein. To obtain plasma, blood samples were centrifuged, aliquoted, and stored at −80 °C until batch-wise analyses. Plasma total cholesterol, high-density lipoprotein cholesterol, triglycerides, and C-reactive protein concentrations were measured on a Roche Modular analyzer (Roche Diagnostics). low-density lipoprotein cholesterol was calculated according to the Friedewald formula. HbA_1c_ was measured with ion-exchange high-performance liquid chromatography (HPLC; Tosoh G8, Sysmex Nederland B.V.). The commercially available protein biomarker panel “Target 96 Inflammation” from Olink proteomics (Olink Bioscience) was used to measure interferon (IFN)-γ (20). Blood samples for RNA isolation were collected in PAXgene Blood RNA tubes (BD Biosciences) and stored at −80 °C following instructions from the manufacturer until batch-wise analyses.

### RNA Isolation and Quantitative Polymerase Chain Reaction Gene Expression Analyses

#### RNA isolation

Total RNA was extracted from whole-blood samples in PAXgene Blood RNA tubes using the automated PAXgene Blood miRNA Kit (PreAnalitiX) procedure, according to the manufacturer's protocol. Briefly, cells were pelleted and lysed. Cell contents were treated with proteinase K and silica-based column extraction was performed, including on-column DNAse I treatment. Total RNA quantity was determined using the Qubit RNA BR Assay Kit (Thermo Fisher Scientific).

#### Complementary DNA synthesis and preamplification

Complementary DNA (cDNA) was synthesized by performing reverse transcription of 50 ng RNA (incubation at 25 °C for 5 minutes, 42 °C for 30 minutes and 85 °C for 5 minutes). Reverse Transcription Master Mix (Fluidigm), containing M-MLV reverse transcriptase, random hexamer, and oligo dT primers, was used. cDNA was preamplified to increase the amount of input material needed for our high-throughput quantitative polymerase chain reaction (qPCR) technique. For preamplification, we used a pool of the target TaqMan assays (Thermo Fisher Scientific, 0.2× each in TE buffer: 10 mM Tris-HCl, 0.1 mM EDTA, pH 8.0) and Preamp Master Mix (Fluidigm) according to the manufacturers’ instructions. Thermal cycling conditions were 95 °C for 2 minutes followed by 14 cycles at 95 °C for 15 seconds and 60 °C for 4 minutes. Preamplified cDNA was diluted 1:5 in TE buffer and stored at −20 °C before analysis.

#### High-throughput quantitative polymerase chain reaction gene expression analysis

mRNA transcripts of 182 genes were measured by high-throughput microfluidic qPCR using 96.96 IFC chips on the Biomark HD system (Fluidigm), as described by the manufacturer. Each TaqMan Assay (20X, FAM-MGB; Supplementary Table S1) (21) was diluted in Assay Loading Reagent (Fluidigm) to a 10× assay mix. Sample mixes were prepared containing 1× TaqMan Universal PCR Master Mix (Thermo Fisher Scientific), 1× Sample Loading Reagent (Fluidigm), and 2.25 μL of preamplified cDNA. The 96.96 IFC chip was primed with Control Line Fluid (Fluidigm) and assay and sample mixes were loaded into the chip using the IFC Controller HX (Fluidigm). qPCR was performed with the Biomark HD using the following thermal cycling protocol: 95 °C for 10 minutes, followed by 40 cycles at 95 °C for 15 seconds and 60 °C for 1 minute. Data were analyzed using Fluidigm Real-Time PCR Analysis Software (version 4.1.3). A cycle threshold (Ct) value less than or equal to 35 was determined as the cutoff for reliable detection. Relative target gene expression was determined by calculating ΔCt using *GAPDH* as the reference gene.

### Ingenuity Pathway Analysis

Ingenuity pathway analysis (IPA; Qiagen) was performed to assess transcriptional regulators and canonical pathways involved in observed differences in mRNA levels of immune genes between ethnicities. For each potential transcriptional regulator, the program calculates an overlap *P* value and activation *z* score. The overlap *P* value is calculated using the Fisher exact test and indicates whether overlap between the genes in the data set and genes regulated by the transcriptional regulator is statistically significant. The activating *z* score quantifies the predicted activation state of a transcriptional regulator. Overlap *P* values less than .01 and *z* scores greater than 2 are considered statistically significant.

### Statistical Analyses

Statistical analyses were conducted using R (version 3.6.2, Team, 2019) and Prism 9 for Windows (version 9.0.1, 2021, GraphPad Software LLC). Normal distribution of the baseline characteristics was tested using the Shapiro-Wilk test. Dependent on whether the data followed normal Gaussian distribution, baseline characteristics between ethnicities and between sexes were compared using a 2-tailed unpaired *t* test or the nonparametric Mann-Whitney *U* test in R. mRNA levels of immune genes were expressed as ΔCT values on logarithmic scale with a base of 2 (log2). Detected proteins were generated as normalized protein expression values on a log2 scale, with larger numbers representing higher protein levels in the sample. mRNA levels of immune genes and protein levels were compared between ethnicities and between sexes using an analysis of variance model (aov) in R with “ethnicity” and/or “sex” as between-subjects factor. For comparisons between ethnicities and/or sex, *P* values were corrected for multiple comparisons using the Benjamini-Hochberg false discovery rate (FDR) in R. An FDR-adjusted *P* value of less than .05 was considered statistically significant. Pearson correlation and simple linear regression were performed in Prism with mRNA levels as the dependent outcome, and IFN-γ or fat mass percentage as independent outcomes. Here, *P* value of less than .05 was considered statistically significant.

Since mRNA levels of immune genes were expressed as ΔCT values on a log2 scale, larger values represent lower mRNA levels in the sample. For the calculation of the log2 fold changes (log2FC) for volcano plots, the mean ΔCT value of Europid patients was subtracted from the mean ΔCT value of Dutch South Asian patients and multiplied by −1 to obtain the correct direction ([Dutch South Asian – Europid] × –1). Relative expression values (= fold changes) used in bar graphs were calculated using the ΔΔCT method, with the Europid patients as the control group. Figures represent geometric mean and geometric standard deviation and all figures were prepared with Prism 9 for Windows (version 9.0.1, 2021, GraphPad Software LLC). All supplemental figures are located in a digital research materials repository (21).

## Results

### Baseline Characteristics

Baseline characteristics are shown in Table 1 and in Supplementary Table S2 (21) for men and women separately. As reported in a previous publication in which the primary end point of the present study was described (22), compared to Dutch Europids, Dutch South Asian participants had a lower BMI (29.4 ± 4.0 vs 32.3 ± 4.0) and lower low-density lipoprotein cholesterol concentration (2.1 ± 0.8 vs 2.6 ± 0.9 mmol/L). In addition, despite being younger (54.7 ± 10.3 vs 59.6 ± 6.5 years), Dutch South Asians had longer diabetes duration (17.4 ± 9.9 vs 10.7 ± 6.4 years) and higher rates of retinopathy (51 vs 9%) and macrovascular diseases (27 vs 5%) than Dutch Europids (22).

**Table 1.** Baseline characteristics

	Dutch Europid (n = 44)	Dutch South Asian (n = 45)
Demographics		
ȃWomen, n, %	19, 43%	27, 60%
ȃAge, y	59.6 ± 6.5	54.6 ± 10.3^*a*^
ȃDiabetes duration, y	10.7 ± 6.4	17.4 ± 9.9^*b*^
Clinical parameters		
ȃWeight, kg	97.0 ± 14.0	79.6 ± 11.9^*c*^
ȃLength, cm	173.2 ± 8.9	165.6 ± 8.8^*c*^
ȃBMI	32.3 ± 4.0	29.4 ± 4.0^*b*^
ȃWaist-height ratio	0.64 ± 0.06	0.61 ± 0.06^*a*^
ȃBody fat, %	37.1 ± 9.4	37.1 ± 9.2
ȃVAT/SAT ratio	0.6 ± 0.3	0.6 ± 0.3
ȃHbA_1c_, mmol/mol	66.1 ± 11.0	67.7 ± 11.5
ȃHbA_1c_, %	8.2 ± 1.0	8.3 ± 1.1
ȃTotal cholesterol, mmol/L	4.8 ± 1.0	4.2 ± 1.0^*b*^
ȃHDL-C, mmol/L	1.2 ± 0.3	1.2 ± 0.3
ȃLDL-C, mmol/L	2.6 ± 0.9	2.1 ± 0.8^*b*^
ȃCRP, mmol/L	3.1 ± 3.3	3.6 ± 4.1
Diabetic complications/comorbidity		
ȃDiabetic retinopathy, n, %	4, 9%	23, 51%^*c*^
ȃDiabetic nephropathy, n, %	11, 25%	10, 22%
ȃDiabetic neuropathy, n, %	15, 34%	12, 27%
ȃMacrovascular disease, n, %	2, 5%	12, 27%^*b*^
Concomitant medication use		
ȃMetformin, mg/d	2047 ± 569	1750 ± 659^*a*^
ȃSulfonylurea, n, %	13, 30%	8, 18%
ȃInsulin, n, %	28, 64%	34, 76%
ȃStatin, n, %	36, 82%	34, 76%
ȃAntihypertensive drug, n, %	34, 77%	32, 71%

Data are presented as mean ± SD unless specified otherwise. Specifically, in the section of diabetic complications/comorbidities, data are presented as the number of patients who reported to have these complications and the percentage of all. *P* values indicate differences between Dutch Europid vs Dutch South Asian patients.

Abbreviations: BMI, body mass index; CRP, C-reactive protein, HbA_1c_, glycated hemoglobin A_1c_; HDL-C, high-density lipoprotein cholesterol; LDL-C, low-density lipoprotein cholesterol; SAT, subcutaneous adipose tissue; VAT, visceral adipose tissue.

*P* less than .05.

*P* less than .01.

*P* less than .001.

### Circulating Messenger RNA Levels of B-Cell Markers and Interferon-Signaling Genes Are Higher in Dutch South Asians Compared With Dutch Europids With Type 2 Diabetes

**Figure 1.**
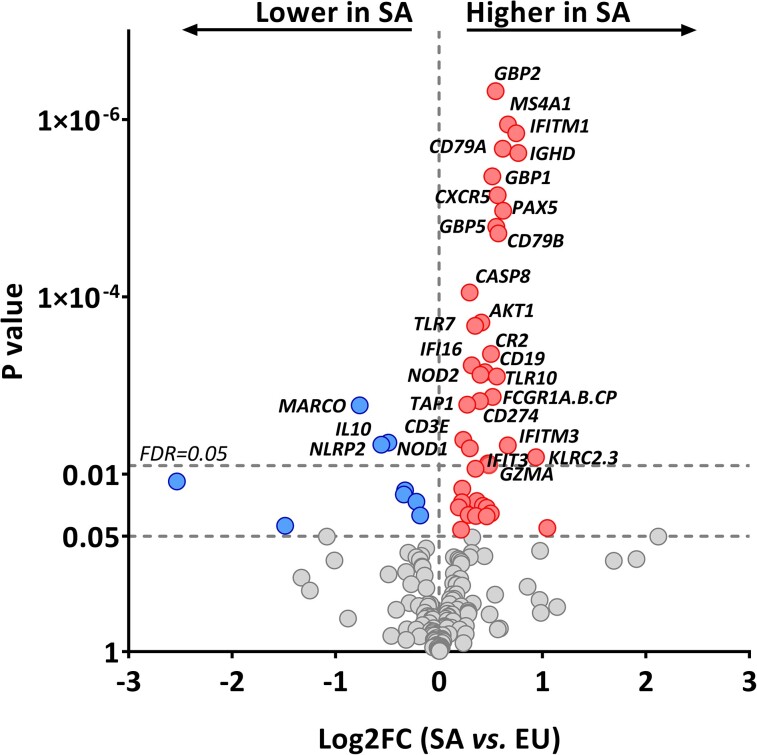
Differences in circulating messenger RNA (mRNA) levels of immune genes between Dutch South Asian vs Dutch Europid patients with type 2 diabetes (T2D). Volcano plot showing the differences in circulating mRNA levels of immune-related genes between Dutch South Asian and Dutch Europid patients with T2D. The x-axis shows the log2FC between Dutch South Asian and Dutch Europid patients, the y-axis shows the *P* value. *P* values were obtained from one-way analysis of variance and thereafter corrected using Benjamini-Hochberg's FDR procedure. The top dashed line represents FDR = 0.05. EU, Dutch Europid patients; FDR, false discovery rate–adjusted *P* value; Log2FC, log2 fold change; SA, Dutch South Asian patients.

**Figure 2.**
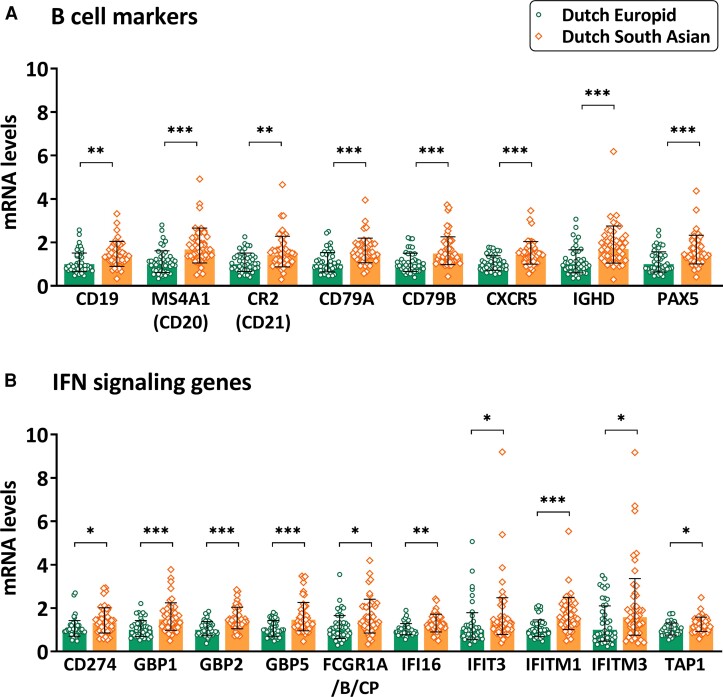
Differences in messenger RNA (mRNA) levels of B-cell markers and interferon (IFN)-signaling genes in Dutch South Asian compared to Dutch Europid patients with type 2 diabetes (T2D). Bar charts showing mRNA levels of A, B-cell markers and B, IFN-signaling genes that are different between Dutch South Asian vs Dutch Europid T2D patients (false discovery rate [FDR] < 0.05). mRNA levels are represented relative to levels of Europids, with geometric mean and geometric SD. *FDR < 0.05, **FDR < 0.01, ***FDR < 0.001 (one-way analysis of variance, thereafter corrected using the Benjamini-Hochberg FDR procedure).

mRNA levels of 30 of 182 (16%) immune-related genes were significantly different (FDR < 0.05) between Dutch South Asian and Dutch Europid participants (Fig. 1). Among those, mRNA levels of 3 genes were significantly lower in Dutch South Asians (scavenger receptor *MARCO,* anti-inflammatory cytokine *IL10,* and inflammasome component *NLRP2*; all FC < 0.7, FDR < 0.04), whereas mRNA levels of 27 genes were higher in Dutch South Asians compared to Dutch Europids. Specifically, mRNA levels of the apoptosis involved *CASP8*, oncogene *AKT1*, T-cell subset marker *CD3E*, natural killer cell marker *KLRC2/3*, cytotoxicity marker *GZMA*, and pattern recognition receptors *TLR7*, *TLR10*, *NOD1*, and *NOD2* were higher in Dutch South Asians than in Dutch Europids (all FC > 1.2, FDR < 0.05). Additionally, we found a clear pattern in which mRNA levels of 8 of 10 (80%) measured B-cell markers (*CD19*, *CD79A*, *CD79B*, *CR2*, *CXCR5*, *IGHD*, *MS4A1*, *PAX5*; all FC > 1.4, FDR < 0.008; Fig. 2A) and 10 of 25 (40%) measured IFN-signaling genes (*CD274*, *FCGR1A/B/CP*, *GBP1*, *GBP2*, *GBP5*, *IFI16*, *IFITM1*, *IFITM3*, *IFIT3*, *TAP1*; all FC > 1.2, FDR < 0.05; Fig. 2B) were higher in Dutch South Asians than in Dutch Europids.

### Interferon-Signaling Pathway Is the top Canonical Pathway That Could Explain Observed Ethnic Difference in Messenger RNA Levels of Immune-Related Genes

**Figure 3.**
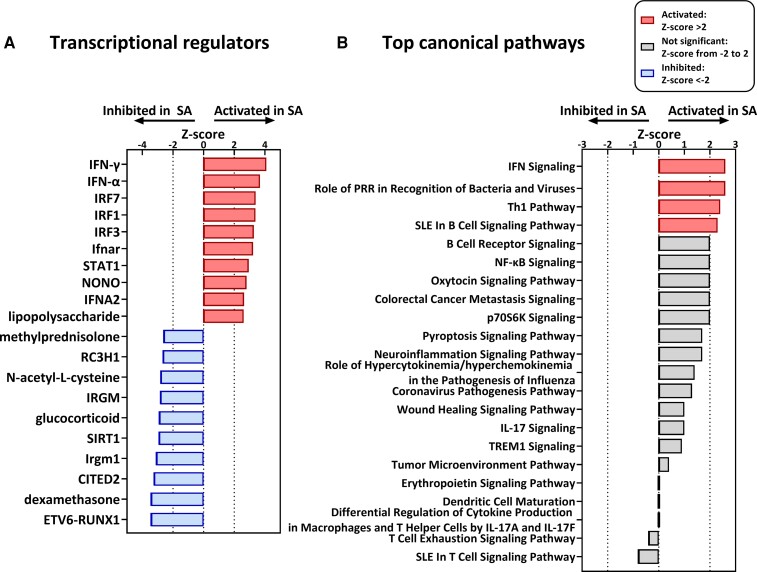
Transcriptional regulators and top canonical pathways that could explain the observed ethnic difference in messenger RNA (mRNA) levels of immune genes. A, The top 10 upregulating and top 10 downregulating transcriptional regulators with a statistically significant overlap (*P* < .01) and a *z* score greater than 2 or less than −2. The y-axis shows the name of the transcriptional regulators; the x-axis shows the *z* score. B, Top canonical pathways with a statistically significant overlap (*P* < .01) and a *z* score greater than or equal to 2. Those with a *z* score greater than 2 appear in red. The y-axis shows the name of the pathway; the x-axis shows the *z* score. Transcriptional regulators and top canonical pathways are obtained from ingenuity pathway analyses, performed on the list of genes with a differential expression (*P* < .05) between Dutch South Asians and Dutch Europids, with as input the log2 fold change differences in mRNA levels.

IPA was performed to identify transcriptional regulators and canonical pathways that could explain the observed ethnic differences in mRNA levels of immune genes. In total, 469 potential transcriptional regulators were identified with a statistically significant overlap (*P* < .01) between the genes in our data set and genes known to be regulated by these transcriptional regulators. Among those, 60 transcriptional regulators had a *z* score greater than 2 or less than −2. The top 20 hits with the highest *z* score are shown in Fig. 3A. IFN-γ had the highest *z* score (*z* score: 4.1; *P* < .001), followed by IFN-α (*z* score: 3.7; *P* < .001), IFN regulatory factor 7 (IRF7; *z* score: 3.4; *P* < .001), IRF1 (*z* score: 3.4; *P* < .001), IRF3 (*z* score: 3.3; *P* < .001), and IFN-α/β receptor (IFNAR; *z* score: 3.2; *P* < .001). In addition, using IPA, 68 significant canonical pathways (*P* < .01) were identified, of which only 4 pathways had a *z* score greater than 2 (Fig. 3B). These top canonical pathways were IFN signaling (*z* score: 2.6; *P* < .001), role of pattern recognition receptors in recognition of bacteria and viruses (*z* score: 2.6; *P* < .001), Th1 pathway (*z* score: 2.4; *P* < .001), and systemic lupus erythematosus in B-cell signaling pathway (*z* score: 2.3; *P* < .001). Thus, both in the transcriptional regulators and canonical pathways, IFN-signaling pathways appeared as a top hit.

Moreover, plasma protein levels of IFN-γ were higher in Dutch South Asians compared to Dutch Europids (FC = 1.5, FDR = 0.01; Supplementary Fig. S1A) (21). IFN-γ protein levels were positively associated with mRNA levels of several IFN-signaling genes that were different between Dutch South Asians and Dutch Europids (Supplementary Fig. S1B) (21). In both ethnicities, IFN-γ protein levels were positively associated with mRNA levels of *CD274*, *GBP1*, *GBP5*, and *IFIT3*. Additionally, in Dutch South Asians only, IFN-γ protein levels were positively associated with mRNA levels of *GBP2* and *FCGR1A/B/CP*, and in Dutch Europids only, with mRNA levels of *IFITM3* (Supplementary Fig. S1B) (21).

### Ethnic Differences in Immune Messenger RNA Levels Are Greater in Women Than in Men

**Figure 4.**
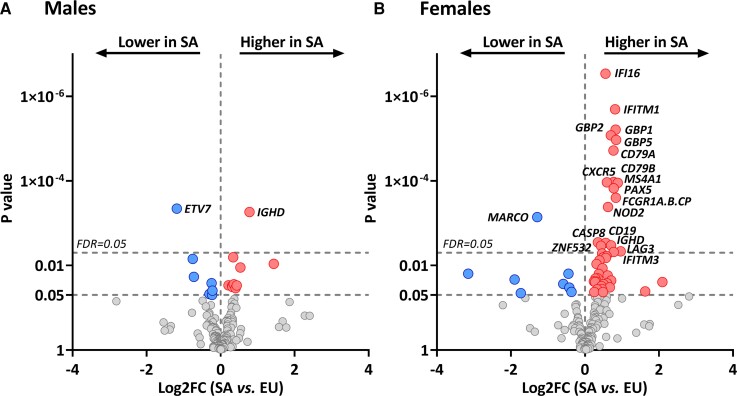
Differences in messenger RNA (mRNA) levels of immune genes between Dutch South Asian vs Dutch Europid males and females with type 2 diabetes (T2D). A and B, Volcano plot showing the differences in mRNA levels of immune-related genes between Dutch South Asian and Dutch Europid T2D patients, in A, men and B, women. The x-axes show the log2FC between Dutch South Asian and Dutch Europid patients; the y-axes show the *P* value. *P* values were obtained from one-way analysis of variance and thereafter corrected using the Benjamini-Hochberg FDR procedure. The top dashed line represents FDR = 0.05. EU, Dutch Europid patients; FDR, false discovery rate–adjusted *P* value; Log2FC, log2 fold change; SA, Dutch South Asian patients.

Interestingly, although we did not observe an interaction between sex and ethnic difference in mRNA levels (*P* > .05), the ethnic difference in mRNA levels of immune genes was more pronounced in women (20/182, 11%) than men (2/182 genes, 1%; Fig. 4A and 4B). In Dutch South Asian males, the mRNA level of *ETV7* was lower than in Europid males (FC = 0.4, FDR = 0.05), and B-cell marker *IGHD* was higher (FC = 1.7, FDR = 0.05; see Fig. 4A). In Dutch South Asian women, the mRNA level of *MARCO* was lower than in Europid women (FC = 0.4, FDR = 0.01), whereas levels of *CASP8*, *NOD2*, *ZNF532*, *LAG3*, and *AKT1* were higher (all FC > 1.3, FDR < 0.05; see Fig. 4B). Moreover, 7 of 10 B-cell markers (*CD19*, *CD79A*, *CD79B*, *CXCR5*, *IGHD*, *MS4A1*, *PAX5*; all FC > 1.5, FDR < 0.04) and 7 of 25 IFN-signaling genes (*GBP1*, *GBP2*, *GBP5*, *IFI16*, *IFITM1*, *IFITM3*, *TAP1*; all FC > 1.5, FDR < 0.05) were higher in Dutch South Asian females (see Fig. 4B).

Next, we performed correlation analyses between baseline characteristics and mRNA levels of B-cell markers, IFN-signaling genes, and IFN-γ protein level, for men and women separately (Table 2). Only a few genes showed an association with baseline characteristics (*P* < .05); however, none of the correlations remained significant after FDR correction for multiple comparisons.

**Table 2.** Associations between baseline characteristics and messenger RNA levels of B-cell markers, interferon-signaling genes, and interferon-γ protein levels

					*Males*										*Females*													
	*Dutch Europid*							*Dutch South Asian*							*Dutch Europid*								*Dutch South Asian*					
	Age	DD	BMI	Body fat %	Waist- height ratio	HbA_1c_	Metf. dose	Age	DD	BMI	Body fat %	Waist- height ratio	HbA_1c_	Metf. dose	Age	DD	BMI	Body fat %	Waist- height ratio	HbA_1c_	Metf. dose	Age	DD	BMI	Body fat %	Waist- height ratio	HbA_1c_	Metf. dose
***B*-*cell markers***																												
*CD19*	−0.14	0.39	−0.03	−0.21	0.07	0.18	0.13	−0.12	0.17	0.12	−0.04	0.10	−0.14	0.17	−0.10	0.27	0.13	0.10	0.19	−0.41	0.11	−0.08	−0.01	0.16	0.11	0.20	−0.25	**0.47*^a^***
*MS4A1* *(CD20)*	−0.12	0.23	0.20	−0.12	0.21	0.03	−0.09	−0.22	−0.03	0.25	0.18	0.14	0.00	0.06	0.13	−0.27	0.30	0.37	0.43	−0.31	0.05	−0.09	−0.05	0.04	−0.06	0.10	−0.26	0.34
*CR2* *(CD21)*	−0.35	0.17	0.01	−0.17	−0.02	0.26	−0.05	−0.13	−0.25	0.19	0.19	0.19	0.05	−0.16	−0.58**	0.17	0.59**	0.22	0.38	0.13	−0.15	−0.26	−0.15	0.15	0.01	0.16	−0.19	0.33
*CD79A*	−0.24	0.24	0.07	−0.21	0.11	0.17	−0.02	−0.12	0.02	0.16	0.12	−0.22	−0.17	0.49	0.12	0.24	0.33	0.28	0.30	**−0.52*^a^***	0.15	−0.11	−0.06	0.11	0.04	0.16	−0.21	0.43
*CD79B*	−0.14	0.28	−0.01	−0.20	0.06	0.11	0.00	−0.07	0.26	−0.23	−0.21	−0.07	−0.09	**0.25*^a^***	−0.02	0.24	0.19	0.03	0.42	−0.44	0.01	−0.09	0.00	0.17	0.11	0.21	−0.11	**0.41*^a^***
*CXCR5*	−0.19	0.33	0.01	−0.15	0.06	0.08	0.04	−0.07	0.15	−0.01	−0.19	0.22	−0.23	−0.09	−0.15	0.17	0.33	0.25	0.24	−0.35	0.05	−0.24	−0.09	0.27	0.15	0.09	−0.21	**0.37*^a^***
*IGHD*	−0.39	0.24	0.15	−0.18	0.09	0.04	−0.05	0.11	0.18	0.12	−0.06	0.21	−0.01	0.09	−0.05	−0.02	0.34	0.25	**0.50*^a^***	−0.41	0.01	−0.19	−0.09	0.28	0.17	0.18	−0.21	0.37
*PAX5*	−0.23	0.24	0.12	−0.15	0.12	0.14	−0.03	0.04	0.25	0.29	0.24	0.36	0	0.19	−0.09	−0.12	0.4	0.37	0.33	−0.32	0.07	−0.1	0.01	0.07	−0.03	0.14	−0.24	0.34
***IFN-signaling genes + IFN-γ***																												
*CD274*	0.21	0.25	0.07	0.08	0.20	0.12	0.28	−0.06	0.25	0.30	0.09	0.27	0.23	−0.40	0.10	−0.08	−0.23	−0.18	−0.18	0.02	0.07	−0.10	0.08	**0.49*^b^***	**0.39*^a^***	0.32	−0.20	0.15
*GBP1*	0.13	0.10	0.09	−0.05	0.03	0.02	0.24	−0.13	−0.07	−0.02	0.02	0.01	0.03	−0.16	0.21	−0.08	−0.07	−0.14	0.15	0.11	0.37	0.01	0.26	0.24	0.03	0.33	−0.23	0.25
*GBP2*	−0.14	−0.16	0.34	0.15	0.30	−0.10	0.03	−0.21	−0.45	0.22	0.14	0.14	−0.05	−0.38	0.13	−0.06	0.03	−0.03	0.26	−0.10	0.01	0.04	0.11	0.25	0.13	0.33	−0.27	0.31
*GBP5*	0.20	0.20	0.11	−0.15	0.06	0.09	0.14	−0.14	−0.13	0.19	0.19	0.17	−0.16	−0.18	0.25	−0.08	−0.18	−0.16	−0.03	0.06	0.19	0.11	0.29	0.30	0.24	**0.48*^a^***	−0.27	0.19
*FCGR1A* */B/CP*	0.03	−0.09	−0.11	−0.04	0.08	−0.13	0.17	−0.04	0.06	0.24	0.20	0.24	0.07	−0.13	−0.22	−0.02	0.18	0.12	0.30	0.00	0.33	−0.25	−0.20	**0.49*^b^***	**0.48*^a^***	0.35	−0.07	0.15
*IFI16*	−0.06	0.13	−0.01	−0.05	0.15	−0.24	0.28	−0.45	−0.29	0.31	0.06	0.13	−0.17	−0.28	0.08	0.05	0.05	−0.29	0.24	0.11	0.29	−0.31	−0.17	0.23	0.13	0.16	−0.19	0.16
*IFIT3*	0.14	0.04	0.03	0.00	0.05	−0.30	0.15	0.26	0.11	0.28	0.44	0.42	0.04	−0.18	0.29	−0.03	0.16	−0.12	0.31	−0.06	0.16	−0.35	−0.15	0.27	0.00	0.19	−0.21	0.09
*IFITM1*	0.01	−0.01	−0.25	−0.24	−0.09	−0.22	−0.05	−0.30	−0.12	−0.07	0.10	−0.12	0.18	0.01	−0.19	−0.03	−0.01	−0.22	0.08	−0.09	0.26	−0.20	−0.07	0.16	0.12	−0.02	−0.32	0.37
*IFITM3*	0.06	−0.01	−0.39	−0.25	−0.24	−0.34	0.11	0.11	0.05	**0.53*^a^***	**0.53*^a^***	**0.58*^a^***	0.26	−0.15	0.13	−0.23	0.35	0.23	**0.54*^a^***	−0.28	0.16	−0.21	−0.12	−0.09	−0.20	−0.31	−0.13	0.21
*TAP1*	−0.06	−0.01	0.23	0.17	0.25	−0.26	0.11	−0.18	**−0.47*^a^***	0.15	0.09	−0.04	−0.45	−0.19	−0.04	**−0.53*^a^***	0.21	0.27	0.18	−0.11	−0.11	0.01	0.04	0.10	−0.01	0.18	−0.25	0.16
*IFN-γ* *(protein)*	0.52**	0.02	−0.24	−0.20	−0.27	0.00	0.18	0.01	0.16	0.20	0.29	0.26	0.21	0.03	0.08	−0.13	−0.10	−0.08	−0.15	−0.07	0.20	−0.08	0.07	0.32	0.29	0.38	−0.38	0.15

Values indicate Pearson *r*. Correlations that were statistically significant before correction using the Benjamini-Hochberg false discovery rate procedure are highlighted in bold (*P* < .05). None of the correlations remained significant after correction.

Abbreviations: BMI, body mass index; DD, diabetes duration; HbA_1c_, glycated hemoglobin A_1c_; IFN, interferon; Metf., metformin.

*P* less than .05.

*P* less than .01.

## Discussion

In this study, we compared mRNA levels of a large panel of immune-related genes between Dutch South Asian and Dutch Europid patients with T2D. We found that mRNA levels of 8 of 10 (80%) measured B-cell markers and 10 of 25 (40%) measured IFN-signaling genes in addition to IFN-γ protein levels were higher in Dutch South Asians compared to Dutch Europids. IPA showed that the IFN signaling pathway was the most activated canonical pathway and IFN-γ the top activating transcriptional regulator explaining these differences. Accordingly, IFN-γ protein levels were higher in Dutch South Asians compared to Dutch Europids. These ethnic differences were more pronounced in women than in men. We hypothesize that an enhanced IFN-signaling pathway may contribute to the more severe disease progression and accelerated risk for T2D-associated complications that is generally found in the South Asian compared to the Europid population.

The finding that the IFN-signaling pathway is more activated in Dutch South Asian compared to Dutch Europid patients with T2D is in seeming contrast with one of our previous findings. In overweight prediabetic men, we found lower mRNA levels of several type I IFN-signaling genes in subcutaneous white adipose tissue and skeletal muscle biopsies of South Asian compared with Europid men, without differences in blood mRNA levels of IFN-signaling genes (23). Although both type I IFNs and IFN-γ (ie, type II IFN) regulate the antiviral response, they are in fact structurally unrelated, bind to a different receptor, and have distinct physiological functions (24, 25). In turn, both studies point toward dysregulated IFN signaling in metabolically compromised Dutch South Asians, perhaps starting with impaired type I IFN signaling in metabolic tissues in individuals with prediabetes, followed by an overall more activated inflammatory response including accelerated IFN-signaling pathways once the disease progresses.

Indeed, the involvement of IFN signaling in the development and progression of T2D has been supported by previous preclinical and clinical studies. In prediabetic obese mice, virally induced IFN-γ (using murine cytomegalovirus) drives the progression from prediabetes to T2D by causing insulin resistance in skeletal muscles through downregulation of the insulin receptor (26). Also in primary human adipocytes, IFN-γ induces insulin resistance by downregulating the insulin receptor, as well as insulin receptor substrate-1 and GLUT4 (27). These preclinical findings are corroborated by an observational study showing that patients with T2D, compared to controls, have higher IFN-γ levels that positively correlate with HbA_1c_ levels (28). Moreover, in a cohort study consisting of 157 overweight Dutch Europid individuals, IFN-stimulated genes were found to be upregulated in the whole-blood transcriptome of insulin-resistant compared with insulin-sensitive individuals (29). These data thus suggest that IFN-signaling pathways may play a role in the pathophysiology of T2D. Unfortunately, in the present study we did not study the severity of peripheral insulin resistance (eg, via hyperinsulinemic-euglycemic clamp). This would have enabled us to study possible associations of IFN signaling genes with insulin resistance. Notably, in patients with T2D, IFN-γ is also shown to be positively related to diabetes-associated complications such as nephropathy (30) and diabetic foot ulcers (31). Thus, a higher activation of the IFN-signaling pathway could contribute to the increased risk of South Asians to develop T2D, but also a more severe T2D progression with a higher risk of T2D-associated complications. Nonetheless, we cannot exclude that a higher activation of the IFN-signaling pathway is reflective of a longer disease progression in South Asian patients with T2D, since the Dutch South Asians included in our study had a longer T2D duration compared to the Dutch Europids.

Next to higher expression of IFN-signaling genes, we found higher mRNA levels of B-cell markers, and lower mRNA levels of interleukin-10 (IL-10), in Dutch South Asian compared to Europid patients with T2D. B cells can contribute to insulin resistance via antigen presentation to T cells, changes in cytokine secretion, and pathogenic antibody production, thereby activating T cells (that secrete, eg, IFN-γ) and polarizing macrophages toward a proinflammatory phenotype (32–34). B-cell–deficient mice on a high-fat diet, compared to wild-type controls on the same diet, display lower blood glucose levels and less insulin resistance (33), accompanied by decreased systemic and adipose tissue inflammation, and decreased adipose tissue IFN-γ expression (34). In healthy individuals, B cells are an important source of anti-inflammatory IL-10 secretion, and circulating IL-10 is positively associated with insulin sensitivity (35). On the other hand, an impaired IL-10 response on a proinflammatory stimulus is related to the presence of metabolic syndrome and T2D in old individuals (36), and B cells from patients with T2D show diminished IL-10 secretion on stimulation of its toll-like receptors (37). In summary, B cells can become involved in inflammation and insulin resistance, among others, by decreasing IL-10 secretion, and activating T cells that produce IFN-γ (32). The results of the present study suggest that this inflammatory pathway is enhanced in Dutch South Asian compared to Europid patients with T2D, and future studies should investigate whether the phenotype of B cells in South Asians indeed associates with enhanced insulin resistance.

We observed that the ethnic differences in immune mRNA levels were more pronounced in female patients with T2D than in male patients with T2D. Generally, premenopausal females are at lower risk of developing cardiometabolic diseases compared to men of the same age. However, after menopause or once T2D has developed, this sex-dependent benefit diminishes and the cardiometabolic risk in women accelerates (38, 39). Consequently, postmenopausal women with T2D are at greater risk of developing cardiovascular comorbidities compared to age-matched men with T2D (39, 40). Interestingly, the age at menopause differs across populations, with lower menopausal ages reported among South Asians (ie, age 44-49 years) compared with Europids (ie, age 50-54 years) (41, 42). In this study, we cannot exclude that more South Asian women have reached the postmenopausal state than Europid women, which, together with their earlier diabetes onset, could result in a more disturbed immune system. Another factor to consider when speculating about the origin of the enhanced circulating mRNA levels of IFN-signaling genes in Dutch South Asian women is adipose tissue. Total body fat percentage was slightly lower in Dutch South Asian women than in Dutch Europid women, and did overall not correlate with mRNA levels of IFN-signaling genes, indicating that total fat mass might not explain the found differences. However, previously it has been reported that healthy young South Asian individuals, compared to Europids, have larger subcutaneous adipocytes (43, 44) and increased adipose tissue macrophage infiltration (45), regardless of total body fat or visceral fat content. Hypertrophic adipocytes that are overloaded with triglycerides are at risk for hypoxia (46). Induction of hypoxia-inducible factor-1 (HIF-1) and endoplasmic reticulum stress cause adipocyte cell death as well as the infiltration of immune cells from the innate and adaptive immune system, such as B cells, T cells, and macrophages resulting in a local and systemic proinflammatory environment (47, 48). Hypothetically, dysfunctional adipose tissue, regardless of adipose tissue mass, may be driving insulin resistance and the progression toward T2D.

One of the strengths of our study is the large array of immune-related genes that were measured both in men and women, allowing us to detect transcriptional regulators and canonical pathways driving the observed differences in mRNA levels. Unfortunately, mRNA levels of immune genes were measured only in whole-blood samples, without isolating cells using flow cytometry analyses, lacking the ability to relate mRNA expression data with immune cell numbers and composition in blood. Furthermore, this could have been followed by ex vivo stimulation experiments on blood cells derived from both ethnicities to study the functionality of immune cells. Moreover, we did not measure inflammatory molecules in insulin target tissues, such as white adipose tissue and skeletal muscle, and as a result we cannot relate differences in circulating immune genes with those in metabolically active tissues. Similarly, we lack information on the menopausal state of women included in the present study. Concerning the study design, it remains a challenge to optimally match Dutch South Asian and Europid individuals. In this study, Dutch South Asians were younger, had a lower BMI, but had a longer diabetes duration, meaning a longer diabetic treatment period, which may have influenced the results. All these mentioned baseline differences can thus be confounders in the present study and results should be interpreted with this in mind. In addition, although medication use did not differ between ethnicities, we cannot exclude that use of prior medication (metformin, sulfonylurea, insulin, antihypertensives, and statins) may have influenced the results. Last, it is important to consider that an ethnicity is defined by cultural traditions. Therefore, we cannot exclude the influence of differences in behavior and lifestyle on the results in this study.

In conclusion, we show that circulating mRNA levels of IFN-signaling genes and B-cell markers are higher in Dutch South Asian than in Europid patients with T2D. We propose that increased inflammation, involving both B cells and IFN-signaling pathways, in Dutch South Asian patients with T2D is possibly contributing to the rapid progression of T2D and its complications in this population. Only future intervention studies can show whether targeting the IFN pathway for the treatment of T2D using anti-inflammatory therapies will be beneficial in the Dutch South Asian population. In this respect, the relatively novel antidiabetics glucagon-like peptide-1 receptor agonists and sodium-glucose cotransporter-2 inhibitors, which have been shown to exert cardiorenal protective effects, are particularly interesting.

## Financial Support

This work was supported by Novo Nordisk A/S (Bagsvaerd, Denmark), the Dutch Diabetes Foundation (No. 2015.81.1808 to M.R.B.), a Maria Zambrano fellowship by the Ministerio de Universidades y la Unión Europea–NextGenerationEU (No. RR_C_2021_04 to B.M.T.), and the Netherlands Cardiovascular Research Initiative: an initiative with support of the Dutch Heart Foundation (No. CVON2017 GENIUS-2 to P.C.N.R.).

## Disclosures

The authors have nothing to disclose.

## Data Availability

Some or all data sets generated during and/or analyzed during the present study are not publicly available but are available from the corresponding author on reasonable request.

## Clinical Trial Information

ClinicalTrials.gov numbers NCT01761318 and NCT02660047 (registered January 4, 2013).

## Abbreviations

BMI: body mass index
cDNA: complementary DNA
FDR: false discovery rate
HbA_1c_: glycated hemoglobin A_1c_
IFN: interferon
IL-10: interleukin-10
log2FC: log2 fold change
IPA: ingenuity pathway analysis
MRI: magnetic resonance imaging
mRNA: messenger RNA
qPCR: quantitative polymerase chain reaction
SAT: subcutaneous adipose tissue
T2D: type 2 diabetes
VAT: visceral adipose tissue
